# Correlation among some phenological and biochemical traits in date palm (*Phoenix dactylifera* L.) germplasm

**DOI:** 10.3389/fpls.2023.1118069

**Published:** 2023-03-28

**Authors:** Riaz Ahmad, Hayssam M. Ali, Anna Lisek, Walid F. A. Mosa, Sezai Ercisli, Muhammad Akbar Anjum

**Affiliations:** ^1^Department of Horticulture, The University of Agriculture, Dera Ismail Khan, Pakistan; ^2^Department of Botany and Microbiology, College of Science, King Saud University, Riyadh, Saudi Arabia; ^3^The National Institute of Horticultural Research, Konstytucji 3 Maja 1/3, Skierniewice, Poland; ^4^Plant Production Department (Horticulture-Pomology), Faculty of Agriculture, Saba Basha, Alexandria University, Alexandria, Egypt; ^5^Department of Horticulture, Faculty of Agriculture, Ataturk University, Erzurum, Türkiye; ^6^Department of Horticulture, Bahauddin Zakariya University, Multan, Pakistan

**Keywords:** biplot analysis, cluster analysis, correlation matrix, genetic diversity, genetic association

## Abstract

Date palm is an economically important fruit crop in the arid and semi-arid regions of the world. The knowledge of genetic variation, genotype-by-traits comparison, as well as the relationship among several phenological traits is effective for developing breeding populations by choosing the best allelic combinations and employing selection approaches. Information regarding the genetic variability and inter-relationships among fruit characteristics is facilitating the plant breeders to efficiently exploit the date palm germplasm for the introduction of elite genotypes. The present study was conducted to examine genetic variation among different date palm cultivars -collected from two different research stations in Pakistan as well as the relationship among their phenological and biochemical characteristics. Biplot analysis revealed that the cultivars ‘Dhakki’, ‘Chohara’, and ‘Kupra’ possessed the highest fruit and pulp weights. Moreover, the fruits of the cultivars ‘Champa Kali’ and ‘Shakri’ had the maximum TSS (total soluble solids) and total sugar content. Similar variations were observed in the mean values of the studied phenological and biochemical characteristics as in the biplot analysis. Cluster analysis on the basis of phenological and biochemical characteristics divided 50 cultivars into seven clusters, showing differences in the studied characters. A correlation matrix revealed that pulp weight had a strong relationship with fruit weight, length, and diameter. A strong correlation also existed among antioxidant capacity, antioxidant activity, and total phenolic content. These results demonstrated a huge diversity in phenological and biochemical characteristics of date palm cultivars as well as a correlation among several characteristics of the studied germplasm, which can be exploited in future breeding programs.

## Introduction

Date palm (*Phoenix dactylifera* L.) belongs to the family *Arecaceae* (Arabnezhad et al., 2012) with probable centers of origin Middle East, Western Asia, India, and Iraq (Ataga et al., 2012). It is a perennial and dioecious fruit tree (Khan et al., 2012). It is an important fruit crop due to its commercial production and cultivation in arid and semi-arid areas of Africa, the Middle East, and South Asia (Kumar et al., 2010). It is extensively cultivated as a major fruit crop in desert areas in Pakistan (Ata et al., 2012). Its fruit has been used as food for more than 6000 years (Shahib and Marshall, 2003). It is an excellent source of carbohydrates, proteins, vitamins, antioxidants, dietary fibers, carotenoids, anthocyanin, and minerals (Tang et al., 2013; Hatami et al., 2023). Thus, it provides high nutritional value for people and is considered a basic part of the human diet in several countries (Mortazavi et al., 2015). These nutritious compounds may vary among the cultivars and depend on the fruit developmental stage as well as the agronomic practices adopted. Its fruit has great potential against chronic diseases, i.e., cardiovascular disease, cancer, diabetes, atherosclerosis,s and neurodegenerative disease (Muralidhara et al., 2017).

Palm date is mostly cultivated through offshoots to ensure the uniformity of cultivars but cross-pollination is one cause of genetic variation in seeded populations and hybrids (Hammadi et al., 2009; Naqvi et al., 2015). Genetic variability among date palm genetic resources has been the basis for the development of high-yielding genotypes. Genetic variation in genotypes enhances the heterozygosity that will further improve crop resistance against biotic and abiotic stresses (Ahmad and Anjum, 2018). These variations also provide allelic variability that might be utilized for the development of new cultivars (Ahmad et al., 2020). The introduction of exotic germplasm, mutation, polyploidy, and hybridization are imperative breeding tools that can be helpful to evaluate superior progenies (Haider et al., 2015). The most important indigenous date palm cultivars are Aseel, Dhakki, and Begum Jungi, and many other local and exotic cultivars are grown in arid and semi-arid areas in Pakistan (Iqbal et al., 2012). The proper description of cultivars through phenological and biochemical traits is imperative because it provides valuable information for further breeding purposes (Pommer, 2012). Usually, genetically diverse parents are selected by plant breeders for hybridization due to greater variability level which provides the path for maximum improvement of the target traits (Akhtar et al., 2014).

Phenological and biochemical characterization, i.e., collecting the necessary information on date palm cultivars, is a prerequisite before starting any breeding strategy for crop improvement programs. Phenological traits are more important for the determination of maturity indices and shelf life of fruits (Awan et al., 2018). These are also very helpful for the sorting, grading, and processing of fruit. Generally, the proper stage for fruit harvesting is the ‘*rutab*’ stage as compared to the ‘*khalal*’ stage, to avoid fruit ripening failure. When fruit is harvested at the ‘*khalal*’ stage, it takes more time to dry and is unable to develop superior fruit quality. Biochemical traits provide nutritional and health benefits that are vital for consumers. There is thus an urgent need to examine the phenological and biochemical traits of date palm germplasm (Ahmad et al., 2020). Accurate information regarding the phenological and biochemical traits of different cultivars is an important factor for better exploitation of the germplasm (Ahmad et al., 2020). The knowledge of genetic variations and their linkage within or among the populations is a prerequisite for better understanding the available genetic inconsistency for further usage in advanced breeding. The description carried out using a huge set of phenological and biochemical traits provides the basis for the evaluation of genetic diversity among date palm genotypes using a multivariate approach (Mehmood et al., 2013).

For germplasm characterization and evaluation, it is necessary that variations among evaluated materials be identified precisely and reliably. In the current scenario, there is an urgent need to identify the phenological and biochemical diversity of fruits for their better utilization and provide essential data for consumers, processors, and exporters. From previous literature, it has been revealed that the phenological and biochemical characterization of the genotypes may also initiate association mapping studies in the future to distinguish the markers linked with economically important traits. Hence, the current study was conducted to highlight the appropriate identification strategies for varietal characterization and registration purposes, and the correction of misnamed date palm genotypes available in Pakistan.

## Materials and methods

### Plant materials

A set of fifty cultivars of date palm was selected from two different research stations in Punjab, Pakistan (**Table 1**). The trees of each cultivar were tagged for two years (2017 and 2018) to collect their fruit. The fruits of these cultivars were harvested at the ‘*rutab*’ stage to record data on different phenological and biochemical traits. The phenological and biochemical data of 20-year-old trees were arranged in a randomized complete block design (RCBD). Geographical and meteorological data from Bahawalpur and Jhang locations are presented in **Table 2**.

**Table 1:** Date palm cultivars collected from different research stations of Punjab, Pakistan.

Genotypes code	Genotypes name	Collection sites
1	Akhrot	Date palm Research Sub-Station, Jhang
2	Dhakki	Date palm Research Sub-Station, Jhang
3	Aseel	Date palm Research Sub-Station, Jhang
4	Hilawi-1	Date palm Research Sub-Station, Jhang
5	Hilawi-2	Date palm Research Sub-Station, Jhang
6	Kantar	Date palm Research Sub-Station, Jhang
7	Makran	Date palm Research Sub-Station, Jhang
8	Chohara	Date palm Research Sub-Station, Jhang
9	Zahidi	Date palm Research Sub-Station, Jhang
10	Burhami	Date palm Research Sub-Station, Jhang
11	Neelum	Date palm Research Sub-Station, Jhang
12	Zarin	Date palm Research Sub-Station, Jhang
13	Haleeni	Date palm Research Sub-Station, Jhang
14	Jaman	Date palm Research Sub-Station, Jhang
15	Kohraba	Date palm Research Sub-Station, Jhang
16	Koznabad	Date palm Research Sub-Station, Jhang
17	Karbalaen	Date palm Research Sub-Station, Jhang
18	Jan Sahr	Date palm Research Sub-Station, Jhang
19	Gokhna	Date palm Research Sub-Station, Jhang
20	Danda	Date palm Research Sub-Station, Jhang
21	Begum Jangi	Date palm Research Sub-Station, Jhang
22	Deglet Noor	Date palm Research Sub-Station, Jhang
23	Peela Dhora	Date palm Research Sub-Station, Jhang
24	Shamran-1	Date palm Research Sub-Station, Jhang
25	Shamran-2	Date palm Research Sub-Station, Jhang
26	Rachna	Date palm Research Sub-Station, Jhang
27	Seib	Date palm Research Sub-Station, Jhang
28	Zardo	Date palm Research Sub-Station, Jhang
29	Shado	Date palm Research Sub-Station, Jhang
30	Peeli Sundar	Date palm Research Sub-Station, Jhang
31	Khudrawi-1	Date palm Research Sub-Station, Jhang
32	Khudrawi-2	Date palm Research Sub-Station, Jhang
33	Wahn Wali	Date palm Research Sub-Station, Jhang
34	Angoor	Date palm Research Sub-Station, Jhang
35	Champa Kali	Date palm Research Sub-Station, Jhang
36	Baidhar	Horticultural Research Station, Bahawalpur
37	Dedhi	Horticultural Research Station, Bahawalpur
38	Sundari	Horticultural Research Station, Bahawalpur
39	Kupra	Horticultural Research Station, Bahawalpur
40	Shakri	Horticultural Research Station, Bahawalpur
41	Eedel Shah	Horticultural Research Station, Bahawalpur
42	Pathri	Horticultural Research Station, Bahawalpur
43	Kur	Horticultural Research Station, Bahawalpur
44	Tarmali	Horticultural Research Station, Bahawalpur
45	Fasli	Horticultural Research Station, Bahawalpur
46	Sufaida	Horticultural Research Station, Bahawalpur
47	Hamin Wali	Horticultural Research Station, Bahawalpur
48	Gajar	Horticultural Research Station, Bahawalpur
49	Halmain	Horticultural Research Station, Bahawalpur
50	Makhi	Horticultural Research Station, Bahawalpur

**Table 2:** Geographical and meteorological data of Bahawalpur and Jhang locations.

Location	Jhang	Bahawalpur
Latitude (°N)	31.2781	29.2272
Longitude (°E)	72.3317	71.3866
Altitude (m)	155	100.28
Maximum temperature (°C)	32.5	35.6
Minimum temperature (°C)	10.2	13.4
Average rainfall (mm)	248	143

### Phenological traits

Twelve fruits were randomly taken from each replication of each cultivar for an evaluation of phenological traits. For this purpose, the descriptors used were as described by Rizk and El Sharabasy (2018). Fruit weight (g), pulp weight (g), and stone weight (g) were measured using a digital weighing balance. Fruit length (mm), fruit diameter (mm), stone length (mm), and stone diameter (mm) were measured using a Vernier caliper.

### Biochemical traits

For biochemical analysis, 40 g of fruit samples were ground in 60 ml of distilled water using a dilution factor. The total soluble solids (°Brix) in the fruit juice were estimated using a hand refractometer. Fruit acidity (%) was determined by using a method described previously (Anjum et al., 2018). Vitamin C content (mg 100 mL^-1^) was calculated from the juice, by the method described by Ruck (1963). Then, 30 µl of fruit juice extract and 2.97 ml of DPPH were mixed in test tubes. The homogenized mixture was kept in the dark for 30 min and an absorbance reading was noted for the estimation of antioxidant capacity. Antioxidant capacity (mM Trolox 100 ml^-1^) and antioxidant activity (%) of fruits were estimated as described earlier (Ozgen et al., 2010). Approximately 1 ml of date juice, 1 ml of Folin-Ciocalteu’s phenol reagent, and 10 ml of sodium carbonate were added to 20 ml of distilled water for estimation of total phenolic content (µg GAE ml^-1^) in fruits as described by Ainsworth and Gillespie (2007). Hortwitz (1980) method was followed for the determination of total sugar content (%).

### Statistical analysis

The phenological and biochemical data were analyzed using the statistical software, Statistix 8.1 (Tallahassee Florida, USA), with three replications for each cultivar. The treatment means were separated using a least significant difference (LSD) test at a 5% probability level. Correlation matrixes were constructed using R statistical software. Biplot analyses were made using XLSTAT, 2023 and dendrograms (Ward linkage, Pearson distance) were constructed using Minitab.

## Results

### Mean values of phenological and biochemical traits

The ‘Chohara’ cultivar showed the highest fruit weight (19.80 g) and pulp weight (18.26 g). ‘Eedel Shah’ had the largest fruit length (48.84 mm) and ‘Kupra’ exhibited the largest fruit diameter (30.37 mm). ‘Dedhi’ showed the highest stone weight (1.74 g). ‘Gajar’ had the largest stone length (31.10 mm) and the largest stone diameter was observed in ‘Kupra’ (30.37 mm) as shown in **Table 3**.

**Table 3:** Variation in phenological traits of 50 date palm cultivars.

Cultivar name	Fruit weight (g)	Pulp weight (g)	Fruit length (mm)	Fruit diameter (mm)	Stone weight (g)	Stone length (mm)	Stone diameter (mm)
Akhrot	12.71 gh	11.72 f	30.05 kl	24.46 de	0.99 j-r	19.21 m-o	10.097 bc
Dhakki	18.99 a	17.74 ab	46.69 a-c	26.17 bc	1.25 c-j	25.35 c-f	8.97 bc
Aseel	9.73 no	8.65 m-o	32.93 h-j	20.33 j-l	1.08 f-p	21.00 i-m	9.62 bc
Hilawi-1	11.17 i-m	10.28 h-k	34.33 gh	15.40 op	0.89 n-s	18.03 op	4.43 e-g
Hilawi-2	11.30 i-l	10.34 g-j	39.50 de	19.95 kl	0.96 k-s	23.45 f-h	9.15 bc
Kantar	11.72 g-i	10.79 f-i	32.09 h-k	23.13 e-g	0.93 l-s	20.02 l-n	9.83 bc
Makran	11.55 h-j	10.40 g-j	33.54 g-j	21.70 g-j	1.150 e-n	22.44 g-k	9.76 bc
Chohara	19.80 a	18.26 a	45.33 bc	25.67 b-d	1.54 ab	26.33 cd	6.33 de
Zahidi	11.40 i-k	10.10 h-l	33.67 g-j	16.47 o	1.30 b-i	18.07 n-p	4.60 e-g
Burhami	9.90 m-o	8.71 m-o	41.37 d	19.23 lm	1.19 d-m	23.40 f-h	8.33 cd
Neelum	11.83 g-i	10.63 f-j	32.20 h-k	16.00 op	1.20 d-l	20.67 k-m	4.33 e-g
Zarin	10.06 l-o	8.70 m-o	17.77 m	12.77 q	1.36 b-f	7.66 q-s	3.33 f-h
Haleeni	7.81 rs	6.97 r-t	14.91 no	9.91 s	0.84 p-s	7.93 q-s	3.67 f-h
Jaman	9.14 o-q	8.04 o-s	32.52 h-j	20.32 j-l	1.10 f-p	21.22 i-l	9.63 bc
Kohraba	18.81 ab	17.71 ab	46.86 a-c	25.70 b-d	1.10 f-p	21.60 h-l	16.30 a
Koznabad	10.18 k-o	8.97 l-o	32.53 h-j	21.60 h-j	1.213 d-k	22.37 g-k	9.33 bc
Karbalaen	7.90 q-s	6.84 st	15.21 no	10.10 rs	1.06 h-p	3.11 u	2.23 h
Jan Sahr	9.96 m-o	8.98 k-o	34.23 gh	20.33 j-l	0.98 j-r	19.23 m-o	9.47 bc
Gokhna	9.87 no	8.34 n-q	33.07 g-j	21.93 g-i	1.53 ab	21.67 h-l	9.77 bc
Danda	9.89 m-o	8.50 m-o	14.17 no	9.17 s	1.39 b-e	9.53 q	4.71 ef
Begum Jangi	4.44 t	3.73 u	14.73 no	9.73 s	0.70 s	8.53 qr	3.99 f-h
Deglet Noor	6.87 s	5.92 t	14.91 no	9.91 s	0.95 k-s	9.48 q	4.69 ef
Peela Dhora	8.24 p-r	6.91 r-t	31.50 jk	19.45 k-m	1.33 b-h	20.05 lm	9.38 bc
Shamran-1	9.34 op	8.19 n-r	15.33 m-o	10.33 rs	1.15 e-n	6.97 rs	2.85 f-h
Shamran-2	9.45 op	8.45 no	39.18 de	20.30 j-l	0.99 j-r	21.63 h-l	8.47 c
Rachna	16.57 cd	15.707 cd	39.80 de	26.70 b	0.86 o-s	22.20 g-k	10.17 bc
Seib	15.40 d-f	13.91 e	37.86 ef	25.17 cd	1.49 a-c	21.14 i-m	10.51 b
Zardo	6.87 s	5.86 t	34.11 g-i	16.77 no	1.02 j-r	22.93 g-i	9.90 bc
Shado	4.17 t	3.06 u	28.10 l	14.57 p	1.11 f-p	22.76 g-j	8.91 bc
Peeli Sundar	15.26 ef	14.23 e	40.41 d	24.39 d-f	1.03 i-q	20.88 j-m	8.96 bc
Khudrawi-1	10.42 j-o	9.50 i-n	15.00 no	10.00 s	0.92 m-s	7.69 q-s	4.00 f-h
Khudrawi-2	10.95 i-n	9.79 h-m	13.93 o	9.60 s	1.16 e-n	7.35 rs	3.67 f-h
Wahn Wali	5.33 t	4.25 u	13.94 o	8.94 s	1.08 g-p	6.72 rs	2.67 gh
Angoor	9.88 m-o	8.75 m-o	16.58 mn	11.52 qr	1.13 e-o	4.60 tu	2.03 h
Champa Kali	12.97 g	11.64 fg	15.43 m-o	10.33 rs	1.33 b-g	7.13 rs	3.50 f-h
Baidhar	7.35 rs	6.29 t	37.55 ef	18.12 mn	1.06g-p	25.57 c-e	9.16 bc
Dedhi	16.26 de	14.53 de	47.03 ab	25.63 b-d	1.74 a	27.13 bc	9.76 bc
Sundari	8.02 q-s	7.14 p-t	35.43 fg	18.10 mn	0.87 o-s	21.56 h-l	8.22 cd
Kupra	19.91 a	18.87 a	44.50 c	30.37 a	1.04 i-q	21.07 i-m	8.87 bc
Shakri	11.31 i-l	9.80 h-m	39.33 de	26.50 bc	1.50 a-c	6.33 st	2.80 f-h
Eedel Shah	17.57 bc	16.64 bc	48.84 a	25.51 b-d	0.94 l-s	25.66 c-e	8.71 bc
Pathri	7.64 rs	6.89 r-t	31.26 jk	19.37 k-m	0.75 rs	20.97 j-m	9.31 bc
Kur	12.22 g-i	10.82 f-h	38.95 de	22.94 f-h	1.40 b-e	24.96 d-f	9.85 bc
Tarmali	7.74 rs	6.90 r-t	34.46 gh	19.77 kl	0.85 p-s	20.20 lm	9.28 bc
Fasli	10.13 k-o	8.68 m-o	37.68 ef	20.85 i-k	1.44 b-d	23.98 e-g	9.24 bc
Sufaida	10.21 k-o	9.38 j-n	40.00 de	21.50 h-j	0.84 p-s	22.17 g-k	8.50 bc
Hamin Wali	8.41 p-r	7.05 q-t	31.67 i-k	22.65 gh	1.36 b-f	21.88 h-l	8.70 bc
Gajar	14.63 f	13.24 e	44.87 bc	22.70 gh	1.39 b-e	31.10 a	8.57 bc
Halmain	10.00 m-o	9.01 k-o	39.47 de	19.90 kl	0.99 j-r	28.68 b	9.42 bc
Makhi	9.17 o-q	8.40 n-p	32.53 h-j	22.47 gh	0.77 q-s	16.66 p	9.76 bc

Mean values sharing different letter(s) in a column are statistically significant at p = 0.05 (LSD Test).

The mean values also confirmed the best-performing cultivars with 18.83°Brix of TSS and 14.36 mg/100 ml of vitamin C content for ‘Champa Kali’, and 18.75°Brix of TSS and 68.81% of total sugar content in ‘Shakri’ (**Table 4**). Moreover, the ‘Neelum’ cultivar demonstrated the highest antioxidant capacity, antioxidant activity, and total phenolic content with values of 70.78 mM Trolox/100 ml, 76.43%, and 73.60 µg GAE/ml, respectively.

**Table 4:** Variation in biochemical traits of 50 date palm cultivars.

Cultivar name	TSS (˚Brix)	Acidity (%)	Vitamin C (mg/100 ml)	Antioxidant capacity (mM Trolox/100 ml)	Antioxidant activity (%)	Total phenolic content (µg GAE/ml)	Total sugars (%)
Akhrot	14.58 n-t	0.03 f-h	7.11 k-n	12.57 q-u	13.56 n-r	13.07 o-v	51.97 i-o
Dhakki	16.33 d-i	0.06 a	9.45 d-k	60.58 b-d	64.52 b-d	62.55 b-d	57.43 d-i
Aseel	15.08 k-p	0.04 b-f	7.11 k-n	27.33 i-m	25.64 i-m	26.48 i-m	47.35 n-r
Hilawi-1	15.67 g-m	0.04 c-g	8.24 f-n	27.94 i-l	26.36 i-l	27.15 i-l	56.89 d-j
Hilawi-2	14.50 o-t	0.03 d-h	7.36 i-n	66.94 a-c	71.98 a-c	69.46 a-c	47.77 n-r
Kantar	14.00 q-u	0.05 a-d	7.89 g-n	68.98 ab	74.37 ab	71.67 ab	48.23 m-r
Makran	15.00 k-q	0.04 a-e	9.93 c-i	31.28 h-k	30.27 h-k	30.78 h-k	52.36 i-o
Chohara	17.08 b-d	0.05 a-d	9.13 e-l	39.39 gh	39.77 gh	39.58 gh	67.90 ab
Zahidi	15.00 k-q	0.05 a-d	9.04 e-l	66.90 a-c	71.94 a-c	69.42 a-c	53.70 h-o
Burhami	14.25 p-u	0.04 c-g	7.54 h-n	55.16 de	58.20 de	56.68 de	26.28 u
Neelum	15.00 k-q	0.03 e-h	7.71 g-n	70.78 a	76.43 a	73.60 a	62.03 b-f
Zarin	13.75 s-u	0.02 gh	7.71 g-n	69.84 ab	75.33 ab	72.583 ab	30.12 u
Haleeni	14.83 l-r	0.03 f-h	6.73 l-n	10.60 r-u	6.20 q-s	8.40 s-v	56.22 e-k
Jaman	13.92 r-u	0.02 h	6.92 k-n	8.37 s-u	3.90 q-s	6.13 s-v	53.54 h-o
Kohraba	15.00 k-q	0.04 b-f	7.18 j-n	38.64 gh	38.88 gh	38.76 gh	51.42 i-o
Koznabad	14.67 m-s	0.03 d-h	8.51 f-m	14.22 o-u	10.32 o-s	12.27 p-v	52.45 h-o
Karbalaen	14.92 l-r	0.03 f-h	7.71 g-n	52.64 d-f	55.26 d-f	53.95 d-f	50.70 j-p
Jan Sahr	13.58 tu	0.020 h	6.03 mn	24.78 j-n	22.66 j-n	23.72 j-n	64.633 a-c
Gokhna	15.08 k-p	0.05 a-c	7.00 k-n	29.99 h-l	28.76 h-l	29.37 h-l	42.06 q-t
Danda	13.25 u	0.03 e-h	6.92 k-n	11.71 r-u	7.38 q-s	9.54 s-v	39.93 st
Begum Jangi	14.83 l-r	0.04 c-g	6.92 k-n	58.89 ce	62.547 c-e	60.720 c-e	39.75 st
Deglet Noor	14.67 m-s	0.04 a-e	7.18 j-n	22.91 k-p	20.33 k-o	21.62 k-q	41.70 r-t
Peela Dhora	14.75 l-s	0.03 f-h	7.71 g-n	27.37 i-m	25.82 i-m	26.59 i-m	42.57 q-t
Shamran-1	14.92 l-r	0.02 gh	9.40 d-l	24.21 k-n	21.99 k-n	23.10 k-o	60.83 c-g
Shamran-2	15.17 j-p	0.04 b-f	13.39 ab	18.07 m-r	14.82 m-q	16.44 m-s	62.70 a-e
Rachna	15.42 i-o	0.050 a-c	13.47 ab	53.18 d-f	55.89 d-f	54.54 d-f	54.47 g-m
Seib	15.33 i-o	0.05 ab	11.26 b-e	7.29 tu	2.47 rs	4.88 uv	55.63 f-l
Zardo	14.75 l-s	0.02 gh	10.90 b-f	14.15 p-u	10.32 o-s	12.23 q-v	53.86 h-n
Shado	15.67 g-m	0.03 f-h	12.50 a-c	6.14 u	1.18 s	3.66 v	44.84 p-s
Peeli Sundar	17.42 bc	0.02 gh	10.10 c-h	16.27 n-t	12.80 n-r	14.54 n-u	37.99 t
Khudrawi-1	15.58 g-n	0.04 a-e	9.84 c-j	21.65 l-q	19.00 l-p	20.33 l-r	50.38 j-p
Khudrawi-2	14.83 l-r	0.04 a-e	12.06 a-d	49.52 ef	51.61 ef	50.56 ef	37.42 t
Wahn Wali	15.00 k-q	0.03 f-h	7.36 i-n	23.82 k-o	21.54 k-o	22.67 k-p	47.28 o-r
Angoor	15.50 h-o	0.03 d-h	7.27 i-n	34.58 hi	34.13 hi	34.36 hi	50.16 k-p
Champa Kali	18.83 a	0.03 d-h	14.360 a	38.35 gh	38.53 gh	38.44 gh	55.28 g-l
Baidhar	16.50 c-h	0.04 b-f	6.03 mn	56.49 de	59.76 de	58.12 de	48.21 m-r
Dedhi	16.75 b-f	0.030 e-h	8.42 f-m	11.71 r-u	7.39 q-s	9.55 s-v	68.52 ab
Sundari	17.17 b-d	0.02 gh	6.92 k-n	10.52 r-u	5.99 q-s	8.26 s-v	64.57 a-c
Kupra	17.67 b	0.04 b-f	9.22 e-l	44.28 fg	45.47 fg	44.88 fg	39.43 st
Shakri	18.75 a	0.04 c-g	8.42 f-m	13.07 q-u	8.97 p-s	11.02 r-v	68.81 a
Eedel Shah	15.67 g-m	0.03 d-h	8.07 g-n	23.81 k-o	22.29 k-n	23.05 k-o	56.88 d-j
Pathri	16.92 b-e	0.03 d-h	8.86 e-l	6.32 u	1.37 s	3.84 v	67.80 ab
Kur	16.58 c-g	0.03 d-h	12.41 a-c	58.68 c-e	62.31 c-e	60.50 c-e	48.37 m-q
Tarmali	15.17 j-p	0.04 b-f	8.86 e-l	38.00 gh	38.13 gh	38.06 gh	42.39 q-t
Fasli	16.00 e-k	0.03 e-h	8.51 f-m	44.39 fg	45.66 fg	45.02 fg	53.66 h-o
Sufaida	15.75 f-l	0.04 c-g	7.27 i-n	27.83 i-l	26.24 i-l	27.03 i-l	58.92 c-h
Hamin Wali	16.25 d-i	0.03 e-h	5.58 n	17.28 n-s	13.65 n-r	15.46 n-t	37.58 t
Gajar	16.17 d-j	0.06 a	7.89 g-n	34.15 h-j	33.63 h-j	33.89 h-j	63.22 a-d
Halmain	16.33 d-i	0.04 b-f	8.33 f-m	7.26 tu	2.45 rs	4.85 uv	49.58 l-p
Makhi	16.33 d-i	0.05 a-c	10.37 c-g	7.54 tu	2.76 rs	5.15 t-v	62.71 a-e

Mean values sharing different letter(s) in a column are statistically significant at p = 0.05 (LSD Test).

### Coefficients of variation of phenological and biochemical traits

Coefficients of variation for seven phenological and biochemical traits were calculated for fifty date palm cultivars (**Table 5**), which showed the levels of variation among all the studied traits. Stone length showed the highest variability (39.95%), while the lowest was observed in stone weight (21.43%). Coefficients of variation revealed higher variability in antioxidant activity (74.35%), while the lowest was recorded for TSS (7.73%).

**Table 5:** Coefficients of variation of phenological and biochemical traits of 50 date palm cultivars.

Variable	Observation	Minimum	Maximum	Mean	SD	CV (%)
Fruit weight	50	4.17	19.91	11.01	3.79	34.42
Pulp weight	50	3.06	18.87	9.88	3.72	37.65
Fruit length	50	13.93	48.84	31.95	10.66	33.36
Fruit width	50	8.94	30.37	18.89	5.85	30.97
Stone weight	50	0.70	1.74	1.12	0.24	21.43
Stone length	50	3.11	31.10	18.40	7.25	39.40
Stone width	50	2.03	16.30	7.56	3.02	39.95
TSS	50	13.25	18.83	15.52	1.20	7.73
Acidity	50	0.02	0.06	0.04	0.01	25.00
Vitamin C	50	5.58	14.36	8.68	2.05	23.62
Antioxidant capacity	50	6.14	70.78	32.13	20.10	62.56
Antioxidant activity	50	1.18	76.43	31.42	23.36	74.35
Total phenolic content	50	3.66	73.60	31.77	21.73	68.40
Total sugars	50	26.28	68.81	51.57	9.95	19.29

SD, standard deviation and CV, coefficient of variation.

### Principal component analysis

The eigenvalue showed that the first four components (fruit weight, pulp weight, fruit length, and fruit diameter) showed the maximum variability among the studied traits of date palm cultivars. However, others showed negligible contribution to the variability of date palm cultivars (**Table 6**).

**Table 6:** Principal component analysis of phenological and biochemical traits of 50 date palm cultivars.

	F1	F2	F3	F4
Eigenvalue	4.727	3.369	1.684	1.007
Variability (%)	33.762	24.061	12.028	7.191
Cumulative %	33.762	57.824	69.851	77.043

### Biplot analyses of phenological and biochemical traits

**Figure 1:**
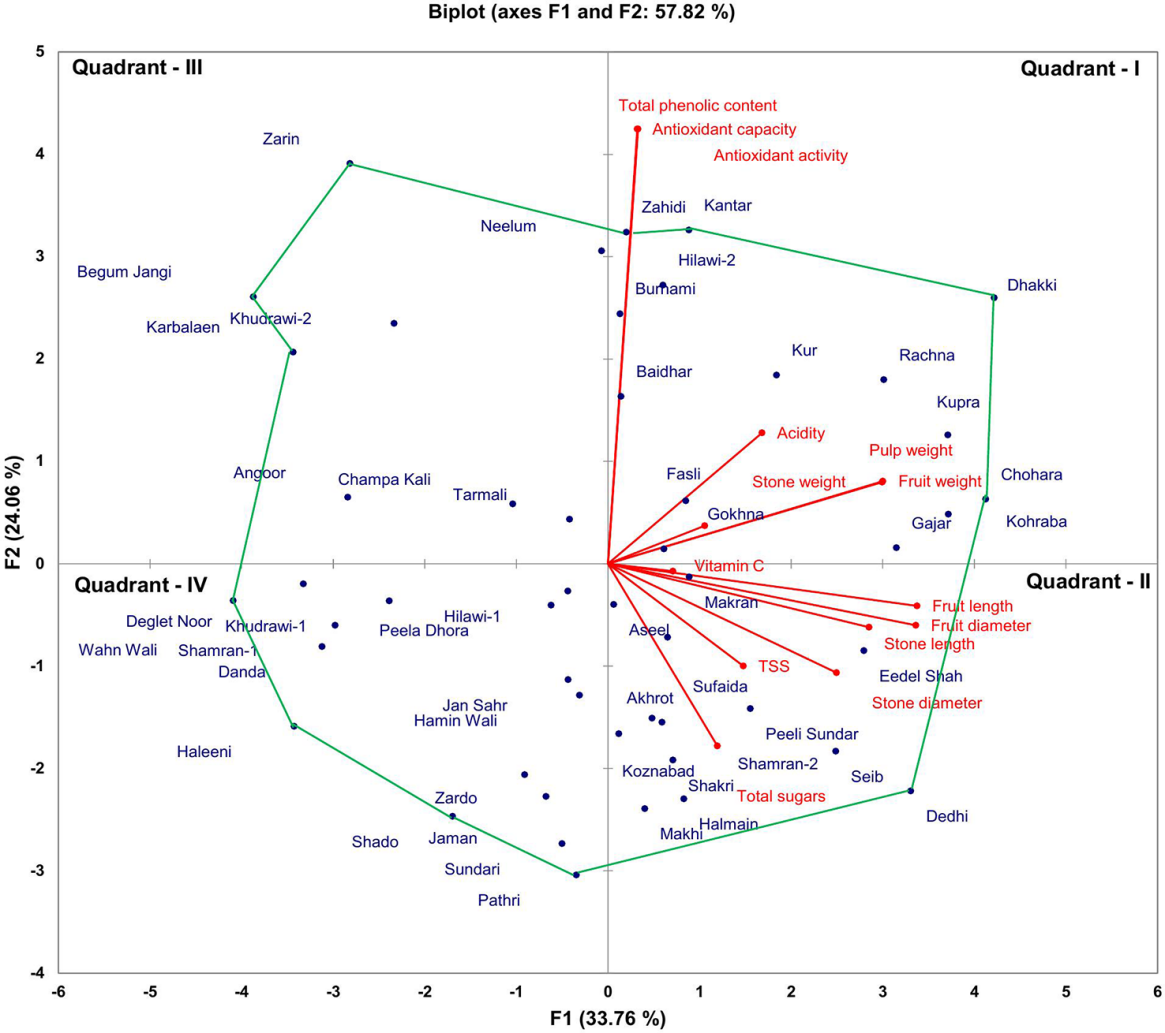
Biplot analysis of pomological and phytochemical characters of 50 date palm cultivars.

Biplot was developed based on the first two components and both components shared a maximum variability of 57.82%. The biplot analysis for phenological biochemical traits revealed that the date palm cultivars, i.e., ‘Dhakki’, ‘Zahidi’, ‘Kantar’, ‘Zarin’, ‘Begum Jangi’, ‘Karbalaen’, ‘Wahn Wali’, ‘Haleeni’, ‘Sundri’, ‘Pathri’, ‘Dedhi’, ‘Kohraba’, and ‘Chohara’, were at the vertex of a polygon. Among these, some genotypes, i.e., ‘Dhakki’, ‘Zahidi’, ‘Kantar’, ‘Dedhi’, ‘Kohraba’, and ‘Chohara’, were found to be more diverse because these were found to be away from the center of the trait vectors. Moreover, genotypes grouped into quadrants I and II showed good performance for the phenological and biochemical traits as compared to quadrants III and IV (**Figure 1**).

### Cluster analysis of genotypes based on phenological and biochemical traits

**Figure 2:**
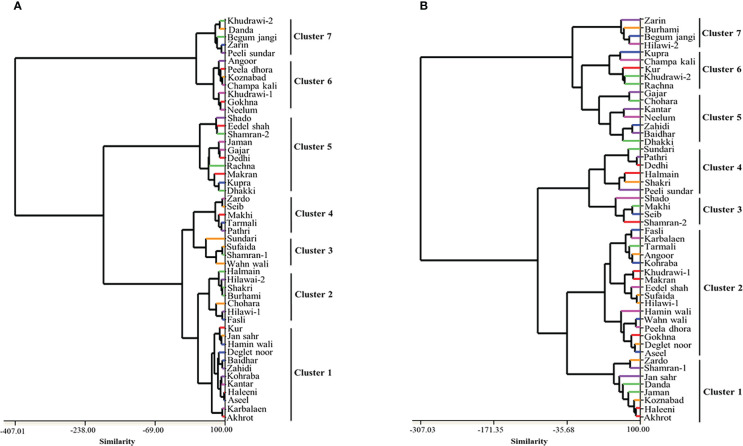
Dendrogram showing relationship among 50 date palm cultivars based on pomological **(A)** and phytochemical **(B)** characters.

A dendrogram based on the phenological traits divided the date palm cultivars into seven major clusters (**Figure 2A**). Cluster 1 comprises a large set of cultivars, i.e., ‘Akhrot’, ‘Karbalaen’, ‘Aseel’, ‘Haleeni’, ‘Kantar’, ‘Kohraba’, ‘Zahidi’, ‘Baidhar’, ‘Deglet Noor’, ‘Hamin Wali’, ‘Jan Sahr’, and ‘Kur’. Two cultivars, ‘Haleeni’ and ‘Aseel’, shared the highest similarity with each other. A dendrogram based on the biochemical traits of the date palm cultivars also grouped the cultivars into seven clusters (**Figure 2B**). Cluster 2 contained a large set of cultivars, i.e., ‘Aseel’, ‘Deglet Noor’, ‘Gokhna’, ‘Peela Dhora’, ‘Wahn Wali’, ‘Hamin Wali’, ‘Hilawai-1’, ‘Sufaida’, ‘Eedel Shah’, ‘Makran’, ‘Khudrawi-1’, ‘Kohraba’, ‘Angoor’, ‘Tarmali’, ‘Karbalaen’, and ‘Fasli’. Two cultivars, ‘Haleeni’ and ‘Akhrot’, were very similar to each other among all the studied cultivars.

### Trait association analysis based on phenological and biochemical traits

**Figure 3:**
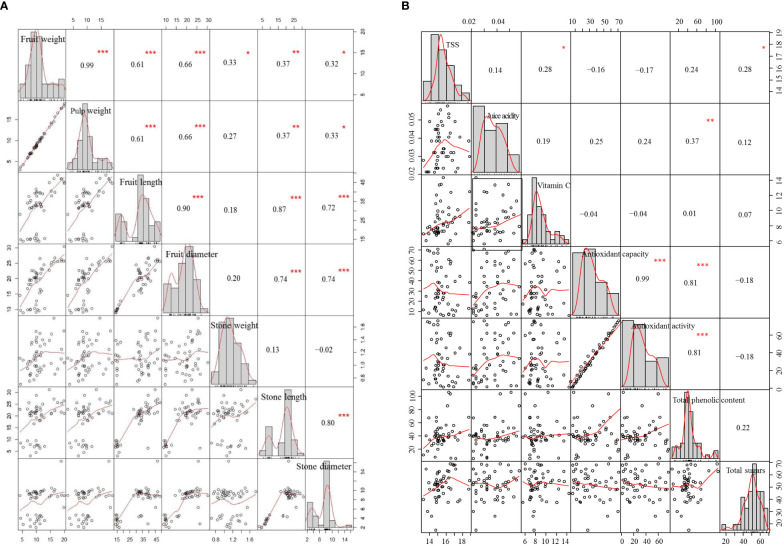
Genotype-by-trait association in pomological **(A)** and phytochemical **(B)** characters of 50 date palm cultivars.

The correlation matrix for phenological traits revealed that fruit weight had a significant association with pulp weight, and fruit length and diameter, while it had a non-significant association with stone weight, length, and diameter. However, stone weight did not show any association with pulp weight, and stone length and diameter (**Figure 3A**). Fruit length showed a significant association with fruit diameter, and stone length and diameter. This association was also confirmed by the biplot analysis as the angle between trait vectors of fruit weight and fruit pulp was less than 90°. Trait association was evaluated through a correlation matrix for biochemical traits (**Figure 3B**). Antioxidant capacity showed a significant association with antioxidant activity and total phenolic content. This association was also confirmed by the biplot analysis as the angle between trait vectors of antioxidant capacity, antioxidant activity, and total phenolic content was less than 90°. The biplot analysis also proved that the correlation matrix is an efficient multivariate tool for the evaluation of trait association among date palm cultivars.

## Discussion

Date palm fruit is considered a good source of carbohydrates, protein, minerals, vitamins, and dietary fiber (Hatami et al., 2023). Genetic variations enhance heterozygosity, which can be exploited to improve crop tolerance against biotic and abiotic stresses. Genetic makeup, climatic conditions, cultural practices, plant nutrition, seed propagation, and cross-pollination are major causes of genetic variability in date palm genotypes. Low crop yield, poor fruit quality, misnaming, and a dioecious nature are major problems for the modern breeding of date palm genotypes (Ashraf et al., 2018). In the present study, readily available date palm germplasm was collected from two research stations in Pakistan. The presence of extensive variation in phenological and biochemical traits depicted ample scope for the characterization of date palm germplasm. Fruit characterization is the foundation for crop evolutionary studies, management of genetic resources, evaluation of the uniqueness of genotypes, and providing basic information for the development of excellent genotypes.

A multivariate approach has been found very effective for the identification of diverse germplasm with desired traits (Wu et al., 2019; Sarikhani et al., 2021). Mean performance and standard deviation can also be utilized for the evaluation of genotypic variation (Faqir et al., 2018). Biplot analysis can be used to identify important traits and genotypes that are the major contributory factors in the variability of date palm germplasm. It is an effective tool for the evaluation of cultivar performance and multidirectional association among different traits (Ennouri et al., 2018). The vertex cultivars in the biplot are those furthest from the biplot origin and these can be excellent or poor in few or all studied traits (Salem et al., 2008). The biplot analysis for phenological traits of the date palm cultivars showed that six cultivars, i.e., ‘Dhakki’, ‘Zahidi’, ‘Kantar’, ‘Dedhi’, ‘Kohraba’, and ‘Chohara’, were at the vertex and were good for phenological and biochemical traits. Biplot analysis depicted that these diverse cultivars for phenological and biochemical traits can be utilized as a source for improvement of elite date palm genotypes and selection breeding might be a suitable approach to bring further improvement in these genotypes or their traits. The mean values of the phenological and biochemical parameters depicted extensive variation in date palm cultivars and this was also confirmed by biplot analysis. This demonstrates that biplot analysis can be used as an alternative tool for the evaluation of genetic variation.

Cluster analysis could be effective for the identification of high yielding genotypes (Faqir et al., 2018). In this study, a dendrogram was constructed which grouped fifty cultivars into seven major clusters based on phenological traits. Two cultivars, ‘Haleeni’ and ‘Aseel’, showed a close genetic relationship as compared to all other studied cultivars and these were grouped together in Cluster 1. The association of studied cultivars for phenological traits during cluster analysis was similar to the biplot analysis. Moreover, cluster analysis also grouped these fifty cultivars into seven major clusters based on biochemical traits. A close relationship was shown between the ‘Sufaida’ and ‘Hilawai-1’ cultivars in Cluster 2 and the highest genetic association was found between the ‘Pathri’ and ‘Dedhi’ cultivars in Cluster 4. The distribution of the fifty date palm cultivars, irrespective of their center of origin, in seven clusters showed that the cluster analysis failed to detect any relationship between genetic divergence and geographical origin. Moreover, the geographical distribution of genotypes is not only the factor that is responsible for genetic diversity. It may possibly be due to genetic drift, artificial selection, climatic conditions, and the exchange of breeding materials. Thus, the selection of parent lines for future breeding purposes might be based on genetics instead of geographical diversity (Salem et al., 2008). Hybridization should be performed among the genotypes of different clusters rather than those of the same cluster to enhance heterosis and desired genetic recombinations (Sharif et al., 2019).

A correlation matrix provides a symmetrical association among a large number of traits (Faqir et al., 2018). A correlation matrix is helpful when an indirect assortment of secondary traits is utilized for the improvement of primary desired traits (Sharif et al., 2019). Fruit weight showed a significant positive correlation with pulp weight, fruit length, fruit diameter, stone weight, stone length, and stone diameter. Antioxidant capacity showed a significant positive correlation with antioxidant activity and total phenolic content. TSS showed a significant association with total sugar content. A similar trait association was revealed through the biplot analysis. These significant positive associations illustrated that all these traits gave similar evidence regarding the differences among genotypes and have a tendency to differentiate the genotypes in a similar manner (Amiri et al., 2010). Among the phenological traits, fruit, pulp, and stone weights were found to be more diverse traits in the studied germplasm as revealed by the mean values and the biplot analysis. Among the biochemical traits, TSS, pH, and vitamin C were found to be more divergent among all the studied germplasm as revealed by the mean values and the biplot analysis. However, cluster analysis is also effective for indicating similarity/dissimilarity among the collected date palm germplasm. A large similarity was recorded in the studied germplasm. Therefore, it is necessary to introduce higher-yielding germplasm for the broadening of the gene pool (Ahmad et al., 2023). It is advocated that great effort, time, and resources might be saved without sacrificing valuable data if an indirect selection is implemented for the improvement of desired traits (Harthi et al., 2015).

## Conclusion

Date palm has diverse germplasm, hence the collection and preservation of this germplasm is a pre-requisite for future crop improvement programs. The characterization of this germplasm is important particularly when evaluated traits are directly related to fruit quality and crop yield. Thus, accurate information on phenological and biochemical traits provides a better guideline for diverse parent selection in breeding programs for the production of ideal products focused on producer and consumer demands. This study demonstrated that large variations exist in the phenological and biochemical traits of date palm cultivars that can be utilized for various crop improvement purposes. Conclusively, the results of the current study might be suitable to manage germplasm collection and helpful for choosing parents in future breeding programs for date palm genotypes.

## Future recommendations

Fruit yield and quality traits are still gaining more attention in the breeding of fruit crops. It has been recommended that phenological and biochemical traits are major points of concern for the identification of higher-yielding elite genotypes. Moreover, mean values, coefficients of variation, biplot analysis, cluster analysis, and trait association are alternative tools for providing the appropriate information for the evaluation of genetic diversity which can be further utilized during diverse parent selection involving new commercial and high-yielding genotypes.

## Data availability statement

The original contributions presented in the study are included in the article/supplementary material. Further inquiries can be directed to the corresponding author.

## Author contributions

RA, HMA and MAA: Conceptualization, Literature review, Writing major and original draft. AL, WFAM and SE: Literature survey, Writing review and editing, Figure designing. All authors contributed to the article and approved the submitted version.
